# An observational study of engineering online education during the COVID-19 pandemic

**DOI:** 10.1371/journal.pone.0250041

**Published:** 2021-04-15

**Authors:** Shadnaz Asgari, Jelena Trajkovic, Mehran Rahmani, Wenlu Zhang, Roger C. Lo, Antonella Sciortino

**Affiliations:** 1 Department of Biomedical Engineering, California State University, Long Beach, California, United States of America; 2 Department of Computer Engineering and Computer Science, California State University, Long Beach, California, United States of America; 3 Department of Civil Engineering and Construction Engineering Management, California State University, Long Beach, California, United States of America; 4 Department of Chemical Engineering, California State University, Long Beach, California, United States of America; 5 College of Engineering, California State University, Long Beach, California, United States of America; KTH Royal Institute of Technology, SWEDEN

## Abstract

The COVID-19 pandemic compelled the global and abrupt conversion of conventional face-to-face instruction to the online format in many educational institutions. Urgent and careful planning is needed to mitigate negative effects of pandemic on engineering education that has been traditionally content-centered, hands-on and design-oriented. To enhance engineering online education during the pandemic, we conducted an observational study at California State University, Long Beach (one of the largest and most diverse four-year university in the U.S.). A total of 110 faculty members and 627 students from six engineering departments participated in surveys and answered quantitative and qualitative questions to highlight the challenges they experienced during the online instruction in Spring 2020. Our results identified various issues that negatively influenced the online engineering education including logistical/technical problems, learning/teaching challenges, privacy and security concerns and lack of sufficient hands-on training. For example, more than half of the students indicated lack of engagement in class, difficulty in maintaining their focus and Zoom fatigue after attending multiple online sessions. A correlation analysis showed that while semi-online asynchronous exams were associated with an increase in the perceived cheating by the instructors, a fully online or open-book/open-note exams had an association with a decrease in instructor’s perception of cheating. To address various identified challenges, we recommended strategies for educational stakeholders (students, faculty and administration) to fill the tools and technology gap and improve online engineering education. These recommendations are practical approaches for many similar institutions around the world and would help improve the learning outcomes of online educations in various engineering subfields. As the pandemic continues, sharing the results of this study with other educators can help with more effective planning and choice of best practices to enhance the efficacy of online engineering education during COVID-19 and post-pandemic.

## 1. Introduction

Engineering education has been traditionally content-centered, hands-on, design-oriented, and focused on the development of critical thinking or problem-solving skills [1]. Various pedagogical methodologies have shown efficacy in enhancement of engineering education including active learning [2], flipped classroom [3] and project-based learning [4–6]. Over the last decade, online education has become a viable component of higher education in engineering subfields such as electrical and computer engineering, computer science and information technology especially at the master’s or post-graduate level [7].

Although the online education has not been a new concept to educators in general, the COVID-19 pandemic introduced an unprecedented and global need to explore online teaching/learning opportunities within the entire spectrum of educational levels and majors. According to the UNESCO, since the onset of pandemic, more than 1.5 billion students worldwide (90.1% of total enrolled learners) have been affected by the COVID-19 closures and subsequent educational changes [8]. The sudden closure of most educational institutions around the world compelled the conversion of the face-to-face instruction into a fully online (or blended/hybrid) format in a short transitional time. As a result, academic institutions that were mainly focused on traditional face-to-face instructions encountered various challenges in this transition [9].

Urgent, careful and evidence-based planning is needed to mitigate the impact of pandemic on engineering education especially for vulnerable, disadvantaged and underrepresented students facing substantial challenges beyond their academic responsibilities, including family obligations, financial burden and additional employments [10–12]. Additional efforts need to be taken to guarantee that the online instruction of engineering courses still meets the rigorous requirements of the program accreditations such as Accreditation Board for Engineering and Technology (ABET).

Despite the existing literature on online engineering education, to the best of our knowledge, there has been no thorough (quantitative and qualitive) analysis of challenges and factors affecting the pandemic online engineering education in the universities that were mainly offering face-to-face classes pre-pandemic. This work is aimed for addressing this gap by considering the following two questions:

1. What are the main challenges influencing online engineering education during COVID-19 pandemic for institutions that were mainly focused on traditional face-to-face instruction pre-COVID?
2. What are the empirical insight and recommendations to address these challenges?

Sloan’s online learning consortium has defined the five pillars of high-quality online education as: learning effectiveness, student satisfaction, faculty satisfaction, access, scale, and cost [1]. Given these factors, we designed and conducted surveys among engineering faculty members and students at California State University, Long Beach (CSULB) to systematically investigate the challenges encountered during the abrupt transition from face-to-face to the online mode of instruction in Spring 2020. This paper presents the results of the conducted surveys and propose solutions for the improvement of online engineering education. Sharing the results of this observational study with other educators can facilitate a more robust continuity of engineering education during ongoing pandemic. It can also aid with overall improvement and consequently further promotion of online engineering education in the post-pandemic era especially for universities that were previously focused on traditional face-to-face instruction. CSULB is one of the most diverse universities in the U.S. in terms of race/ethnicity, gender, financial and cultural characteristics (e.g. with a large percentage of first-generation or low-income students). Thus, the results of this study can especially help the institutions with similar demographics to enhance their online engineering education during and post-pandemic.

### 1.1. Related work

The existing literature has identified several challenges that need to be considered for the effective design and offering of online courses:

- Converting a course from conventional face-to-face to the online format is time consuming and requires the instructor’s familiarity with (or willingness to learn about) online learning pedagogy and instructional tools, including the learning management system (LMS) [13].
- Some students prefer to learn difficult concepts face-to-face [14] and believe that face-to-face instructions provide deeper level of learning compared to the online [15].
- Designing a fair, equitable, and rigorous assessment to minimize cheating and plagiarism is difficult in online environment [16].
- A successful education requires creating and maintaining a reliable and robust infrastructure that supports both faculty and students [7, 17–19].
- Hands-on training to work with equipment, instruments, and materials in a controlled laboratory setting is an inherent and necessary aspect of a successful engineering education [1, 10]. Addressing this essential aspect within a fully online teaching platform is challenging particularly at the undergraduate level.

Recently, several studies have tried to identify the major factors and best practices that contribute to the acceptance, assimilation and success of online education including course design, course content support, instructor’s personal characteristics and students’ familiarity with and access to technical resources [20–22]. Due to sudden conversion to online instructions, caused be COVID-19, faculty and students at academic institutions, mainly focused on traditional face-to-face instruction, encountered various challenges. As the pandemic continues, a small body of literature on educational impact of COVID-19 is starting to emerge. A group of investigators conducted a U.S. nationwide survey study among faculty and students of STEM fields in June 2020. Their results highlighted the gender disparities in online learning during pandemic: female faculty and students reported more challenges in technological issues and adapting to remote learning compared with their male peers [12].They also found out that 35.5% of doctoral students, 18.0% of master’s students and 7.6% of undergraduate students would have a delayed graduation due to pandemic [11]. Hispanic and Black undergraduates were two times and 1.7 times more likely, respectively, to delay graduation relative to Whites.

Dhawan presented a comprehensive *literature review* on the existing pedagogical approaches for the online instruction while identifying the strengths, weaknesses and challenges of adopting each approach for the online education during the COVID-19 pandemic [9].

Vielma and Brey conducted a *qualitative* surveying from 170 students who took asynchronous classes within two engineering departments (biomedical engineering and chemical engineering) at a U.S. Hispanic-serving institution [10]. The goal was to assess the effectiveness of their online education during pandemic. Their results indicated the students’ need in having synchronous instructional content (in addition to asynchronous content) to enhance the social component of learning.

Almaiah et al. conducted a *semi-structured interview* (using a list of general topics as interview guideline instead of a structured list of questions) with 30 students and 31 experts in the field of information technology from six universities in Jordan and Saudi Arabia. Their goal was to identify the challenges that impede the successful employment of online education during pandemic in developing countries and provide educational stakeholders with useful guidelines to enhance education efficacy.

Our work conducts a thorough (*quantitative* and *qualitive*) analysis of challenges and factors affecting the online education of *engineering* courses by conducting surveys among *students* and *faculty members* from *various engineering subfields* at one of the *largest and most diverse* four-year U.S universities (CSULB). Thus, the presented work has several unique aspects that distinguish it from the few existing studies focused on online education during pandemic, such as the use of both quantitative and qualitative survey questions, and participation of large number of engineering students and faculty from various subfields and diverse backgrounds. Our observational study provides empirical evidence for various solutions we propose to enhance online engineering education during and post-pandemic, especially for those universities with limited resources, or with a large population of minority, first-generation and low-income students.

## 2. Materials and methods

### 2.1. Engineering education at CSULB

California State University, Long Beach (CSULB) is one of the largest and most diverse four-year universities in the U.S. Approximately 52% of CSULB student body are NSF-defined underrepresented minority including 59.2% female, 46.9% Hispanic, 4.5% African American and 1% Native American [23]. As a result, CSULB is recognized as a minority serving institution: namely Hispanic, Asian American, Native American, and Pacific Islander-Serving Institution. Also, more than half of our students are low-income or first-generation college students. CSULB College of Engineering (COE) currently has more than 250 faculty and 5000 students (undergraduate and graduate). COE offers a total of 11 programs that are hosted by six departments: Biomedical Engineering (BME), Chemical Engineering (CHE), Civil Engineering & Construction Engineering Management (CECEM), Computer Engineering & Computer Science (CECS), Electrical Engineering (EE), and Mechanical & Aerospace Engineering (MAE). The majority of the courses in COE were offered face-to-face prior to pandemic. Since 2010, CSULB has been using an LMS called BeachBoard (BB) – a customized version of "Brightspace" platform developed and supported by "Desire 2 Learn" company. BB provides various features to facilitate the course instruction, including a robust platform for communication between the instructor and students, sharing course materials and recorded lectures with students, discussion forums, design and management of assessments, assignments and grades. Prior to pandemic, while some CSULB faculty members had been employing (at least some of) BB features (e.g. gradebook) for their instruction on a regular basis, many others had opted out as its usage has not been mandatory.

The unprecedented circumstances of global COVID-19 pandemic forced the swift conversion of the mode of instruction from face-to-face to fully online for all CSULB engineering programs (including 349 courses for a total of 1004 sections) within a transitional period of 10 days in March 2020. Hence, the teaching materials and assessment methods had to be developed “on the fly”. CSULB advised instructors to mainly focus on learning/using BB (and Zoom videoconferencing) to convert their instructions to the online format. This recommendation seemed reasonable given the availability and practicality of BB features. However, both our students and faculty encountered various challenges during the online instruction in Spring 2020. By the end of the semester in May 2020, CSULB announced that Fall 2020 semester was also going to be in the alternative mode of instructions. Thus, 313 engineering courses were scheduled to be offered in synchronous fully online format. 18 additional classes were exempted and offered in hybrid/blended format. These were the classes where the face-to-face component is considered essential to meet the course learning outcomes and therefore could not be conducted fully online, (e.g. laboratories and senior design capstone projects).

### 2.2. Surveys

Our goal was to identify and study the magnitude of various issues that our faculty and students encountered during the six weeks of online instruction in Spring 2020 (March 23-May 8) and plan for an enhanced online instruction in Fall 2020. The faculty and student surveys were designed holistically considering the overall verbal feedback received from stakeholders during the Spring 2020 online instruction. The faculty survey consisted of 10 multiple-choice and 2 free-response questions, while student survey included 8 multiple-choice questions with fill-in or additional comment options for each question.

The faculty survey questions covered a variety of online teaching issues including, but not limited to, the lack of access to necessary hardware (e.g. computer/tablet, stylus, scanner/printer, microphone/headset, camera), software and reliable internet connection. Some questions focused on various learning assessment methods that instructors used in Spring 2020 (or the ones they were planning to use in Fall 2020) including open-book or closed-book exams, synchronous or asynchronous exams, fully-online exam (using randomized questions on BB) or semi-online exams (where students solve the assigned problems on a paper, then scan and upload their solutions on BB). Some questions focused on proctoring exams and the instructors’ perceived prevalence of cheating/plagiarism. Faculty were also asked to indicate the topics that they were interested to enhance their skills on, e.g., basic or advanced BB features, Zoom features, automatic grading, etc. The two open-ended questions provided instructors additional opportunities to comment about their online teaching experience and make any suggestion or request to COE that could help with improvement of online instruction in Fall 2020.

The student survey was designed to identify the challenges students confronted during online instruction in Spring 2020, including lack of access to hardware, software, reliable internet connection, quiet/private space to study, potential issues of balancing study with work and family duties, and stress management. The students were also asked about the difficulties they had during the synchronous classes on Zoom (e.g., lack of focus or engagement, instructor’s lack of familiarity with technology) or during the online exams (e.g., time management, issues with methods of proctoring using camera). Copies of faculty and student surveys are enclosed in the S1 Appendix for the readers’ further reference.

## 3. Results

The faculty survey was conducted using Qualtrics over a three-week period (June 20-July 10). Similarly, the student survey was designed and conducted in Qualtrics afterwards (July 27-August 12). This later timeframe was decided based on the assumption that more students (including the incoming students) might be available to participate in the survey closer to the beginning of the Fall 2020 semester (August 21). Participation in both surveys were anonymous.

**Fig 1:**
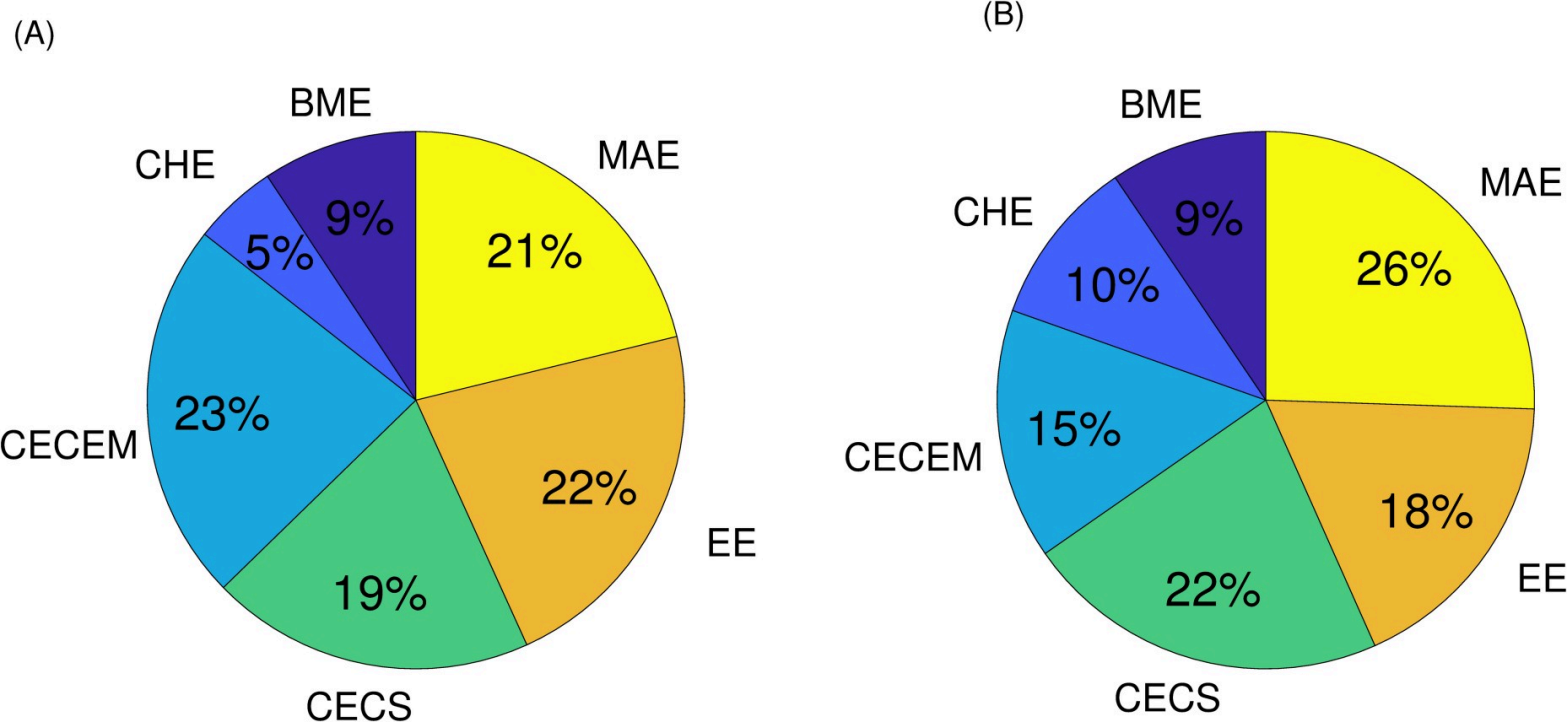
Distribution of the survey participants among various departments within the college of Engineering: (A) Faculty participants; (B) Student participants.

A total of 110 instructors took the survey where 43% of them were full-time including tenured/tenure track faculties and the rest were part-time lecturers. Also, 627 students participated in the survey: First-year students (4%), Sophomore (14%), Junior (30%), Seniors (35%) and graduate students (17%). Fig 1 shows the distribution of survey participants among various departments within the COE (question #1 on both surveys). We observe that all departments have relatively similar representations in terms of percentage of faculty and student participants in respective surveys (9% BME, 5–10% CHE, 15–23% CECEM, 19–22% CECS, 18–22% EE, and 21–26% MAE).

These percentages are consistent with the size of our departments in terms of total number of faculty and students. Therefore, our survey sample population could be a good representative of the general COE populations in terms of existing majors.

### 3.1. Logistical challenges for both students and faculty

**Fig 2:**
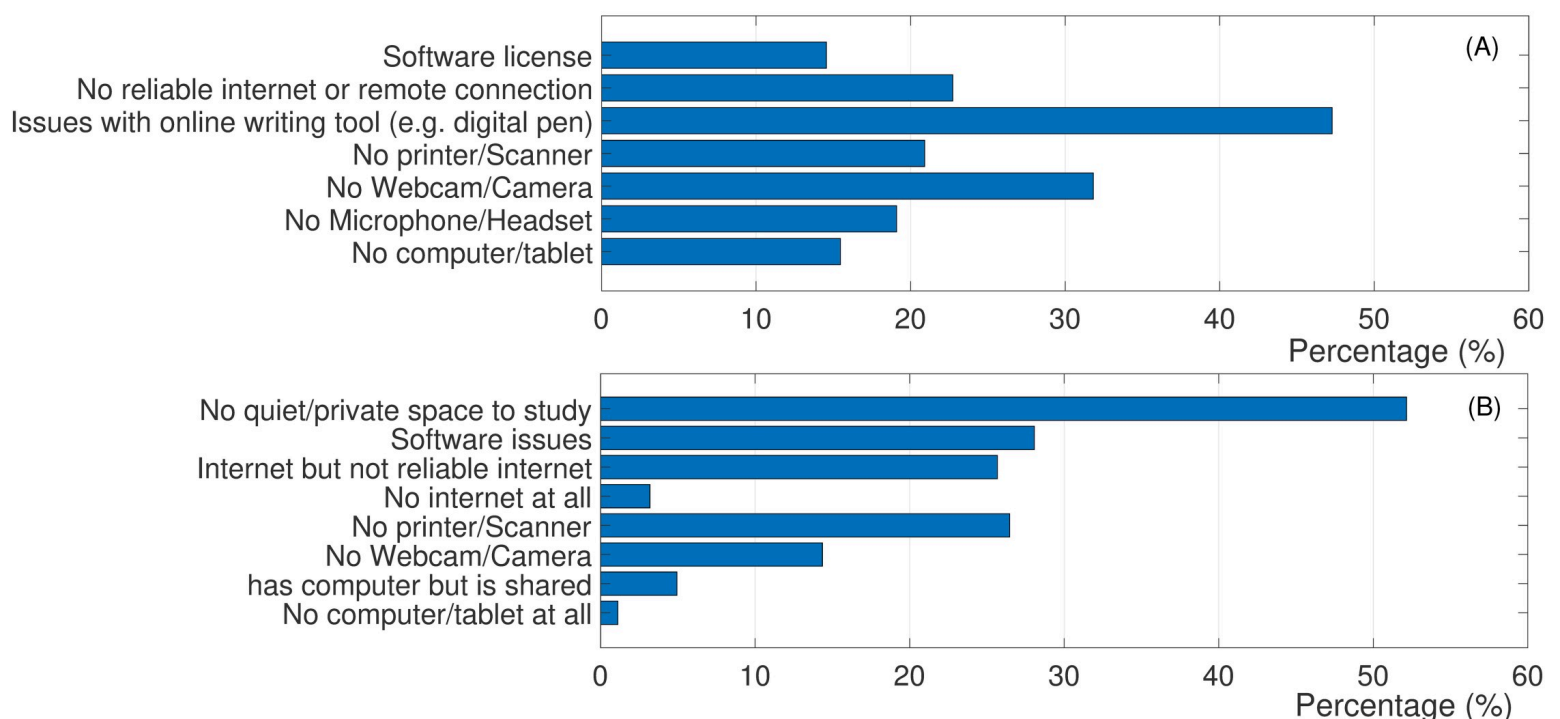
Logistical challenges of online instruction from perspectives of faculty and students. The horizontal access represents the percentage of survey participants who indicated the corresponding challenge. (A) Faculty respondents; (B) Student respondents.

Fig 2 shows the percentages of survey respondents who indicated various logistical challenges they had during the online instruction period of Spring 2020 (question #3 on the faculty survey and question #3 on the student survey). Close to 15% of the faculty had issues with software license or no access to personal computer/tablet. About 20% of the faculty did not have access to microphone/headset or printer/scanner. 23% of faculty had no reliable internet connection, while 32% had no access to webcam or camera for the online instruction. Finally, 47% of the faculty indicated that they had no access to or had technical difficulties with online writing tools. Among the student respondents, 1% had no access to any computer/tablet, while close to 5% had only access to a shared computer at home. 3% had no internet connection, while 26% had issues with reliability of their internet. 28% indicated having issues with software access, while 26% had no printer/scanner at home.

### 3.2. Students challenges with online instruction

**Fig 3:**
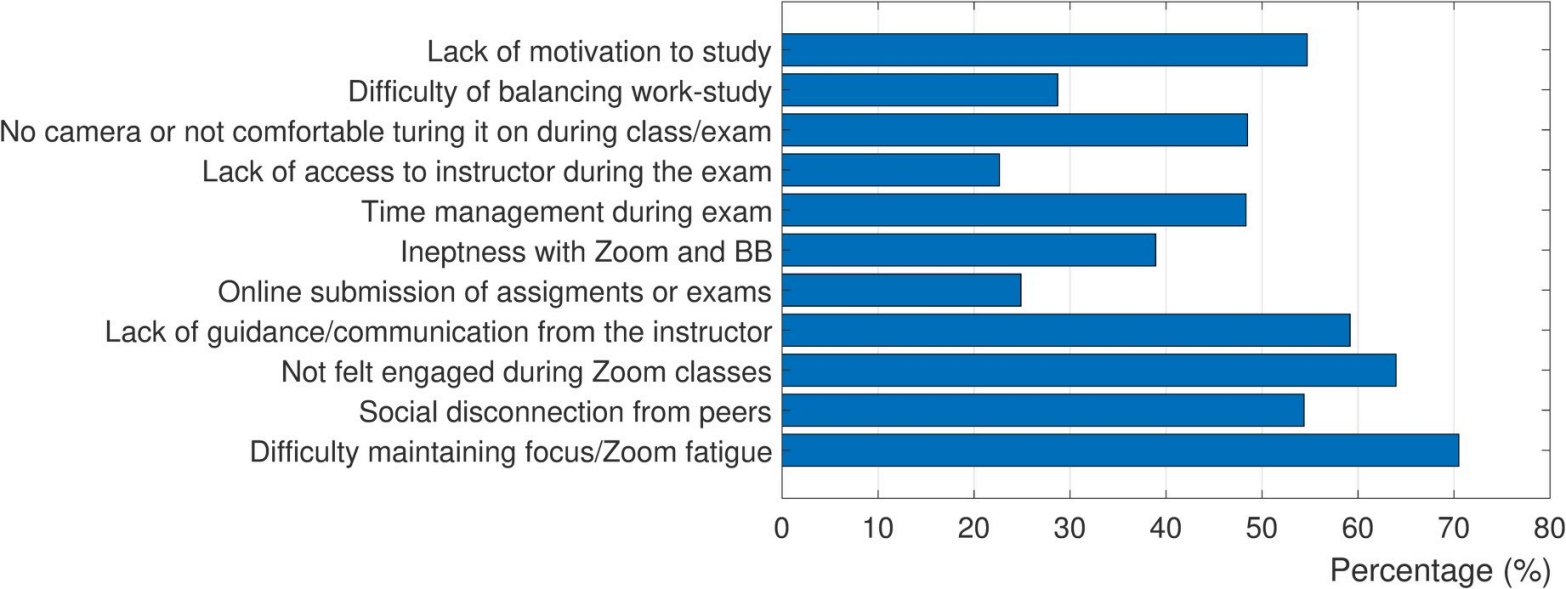
Prevalence of challenges students encountered during online instruction in Spring 2020.

Fig 3 summarizes the prevalence of challenges students had with online instruction during Spring 2020 (questions # 3–6 on the student survey). About 70% of students indicated difficulty in maintaining their focus or experiencing Zoom fatigue after attending multiple online sessions. 55% of students felt social disconnection from their classmates/peers, while 64% did not feel engaged during the online classes. 60% of the students felt there was a lack of clear guidance or communication from the instructors. Also, a quarter of students had issues with online submission of assignments and exams, mainly due to the lack of access to printer/scanner as we learned from students’ optional comments. About 40% of students had technical difficulty and ineptness issues with using or navigating through Zoom or BB. 48% of the students experienced time management issues during the online exams. In optional comments, some students expressed their frustration with not being able to go back to previous questions (a BB feature for the instructors to limit cheating). 23% of the students indicated that the unavailability of the instructor during the online exam (in contrast to in-person exam) caused challenges.

48% of the students specified that they either do not have camera or feel uncomfortable turning the camera/microphone on during the class or online exams (question #7 on the student survey). Optional comments revealed that many participants have privacy concerns with usage of camera/microphone or being recorded, especially if they were living in a crowded home or shared space. Furthermore, some students experienced an increased level of anxiety being watched on camera that hindered their focus and lowered their performance during the online exams. 28% of the students indicated that they had difficulty with balancing work and study. From the optional comments, we understood that the latter issue has been escalated for many during pandemic. Some parents had lost their jobs and consequently the whole family was relying on the part-time jobs of the younger adults (students) to survive financially.

Our survey also indicated that more than 50% of our students did not have access to a private or quiet space to attend the online classes or to study. 55% of students also lacked motivation to study (question #3 on the student survey). The optional comments shed further light onto the lack of motivation: the uncertainty of the COVID-19 pandemic and loss of peer interaction/support were identified as the major contributing factors. Finally, 24% of the students rated their overall experience of online instruction (question #8 on the student survey) as satisfying, 37% found it dissatisfying, while the rest (39%) were neutral.

### 3.3. Assessment methods used during emergency online instruction

Table 1 shows the prevalence of various methods that the faculty used to assess students’ learning during the online instruction of Spring 2020. Semi-online refers to an exam where students solve the assigned problems on a paper, then scan and upload their solutions. Asynchronous exam refers to a take-home exam while a synchronous exam is the one conducted during the scheduled class or exam time. The survey allowed respondents to choose more than one assessment method per question (because faculty might have taught multiple classes, held more than one exam during the semester or applied multiple assessment methods in the same class), thus the sum of the percentages would not equal to 100.

**Table 1:** Learning assessment methods faculty used during the online instruction in Spring 2020. The respondents could choose more than one option for each question depending on the number of exams administered during the semester.

Survey Question #	Assessment method	Percentage of faculty who employed the method
Question #4	Fully online exam (e.g., BB quiz)	63%
	Semi-online Asynchronous exam	28%
	Semi-online Synchronous exam	40%
	Project/term paper	50%
	Oral presentation/demo	33%
Question #7	Open-book/Open-note exam	70%
	Closed-book/Closed-note exam	33%

We observe that the fully online exams such as the BB quizzes were used by 63% of the faculty. BB quizzes provides the faculty with the convenient option of randomizing the order and/or the parameter values of the questions. The instructor can also limit the view to one question per page for students and prevent them from going back to previous questions. The effectiveness of these options in limiting cheating/ plagiarism, and consequently the reduced need for further proctoring, might have contributed to the high popularity of this assessment method among the faculty.

The remaining assessment methods in the decreasing order of their prevalence were project/term paper (50%), semi-online synchronous exam (40%), oral presentation/exam (33%), and semi-online asynchronous exam (28%). Our survey also revealed that 70% of the faculty used the open-book/open-note exam while 33% tried closed-book/closed note exams. The preference of open-book/open-note exam among faculty could be also justified by the decreased need for proctoring tools. In fact, our data (faculty survey question #7) revealed that among those faculty who employed open-book/open-note exam, only 27% used Zoom camera and microphone for proctoring of the exam. 21% used lockdown browsers (e.g. respondus), while 61% did not have any proctoring. However, when the exams were closed-book/closed-note, 56% of the faculty decided to proctor the exam using Zoom camera and microphone, 18% chose to use the lockdown browsers and 35% did not proctor. We also evaluated the association of instructors’ perception of cheating/plagiarism with various assessment methods by calculating the Pearson correlation of faculty’s assessment methods with their trichotomized perception of online cheating (less cheating, the same, more cheating) relative to that of face-to-face (faculty survey question #9). The results revealed no statistically significant correlation between perception of cheating and assessment methods except for the following: Semi-online asynchronous exam (correlation = 0.23, p-value = 0.01) and Closed-note/Closed-book (correlation = 0.21, p-value = 0.03). This data analysis shows that semi-online asynchronous and closed-book exams were associated with an increase in the perceived cheating,

### 3.4. Perceived faculty skills that needed enhancement

Faculty indicated various topics that they were interested to enhance their skills in, as summarized in Table 2.

**Table 2:** A list of topics identified by faculty for further skill enhancement. Respondents could choose as many topics as they were interested to learn.

Survey Question #	Topics	Percentage of faculty interested
Question #10	The major requirements of syllabus for an online course	38%
	Basic BB features: How to create/modify/improve BB for my course	26%
	More advanced BB features: How to create online surveys /discussion groups/quizzes that reduce the potential of cheating, how to automatically export grades to BB gradebook, how to use Master Shell in BB, etc.	58%
	Zoom features (basic and advanced): How to schedule/record a meeting, how to use Zoom’s Whiteboard or OneNote, how to do breakout rooms, etc.	39%
	Multimedia skills: How to create interactive multimedia files using Kaltura Capture, Camtasia or Snagit, how to use Alt captions in media you generate (Word, PPT, page in BB) to facilitate accessibility	39%
	Assessment: How to use automatic grading tools (e.g. Gradescope)	54%

About 60% of the faculty needed to learn about the advanced features of BB (e.g. how to create online surveys or make quizzes with randomized questions or personalized parameter values). Also, more than half of the faculty were interested in learning about semi-automatic grading tools (e.g. Gradescope). Close to 40% of the faculty needed to learn how to create a syllabus for an online class or become more competent with using Zoom features. A similar percentage of participants indicated interest in enhancing their multimedia skills (e.g. working with Kaltura Capture, Camtasia or Snagit). Finally, 26% of the faculty needed more training to become familiar with basic features of BB. In the optional comments (faculty survey questions #11–12), some faculty members expressed their concerns about the delivery of the hands-on components of their courses and requested some general guideline on how to address this issue for an online instruction.

## 4. Discussion

In this section, we will discuss the challenges we identified and propose relevant interventions to improve the online delivery of engineering courses during pandemic.

### 4.1. Student challenges

Our results revealed that a quarter of our students did not have access to reliable internet connection, triggering a concern about widening of the digital equity gap among students due to COVID-19 pandemic. With COVID-19 and the abrupt transition to online teaching, access to reliable internet connection and personal computer/tablet have become major factors affecting the learning outcomes for students. To address this issue, institution can provide WiFi access on campus’s open areas and well-ventilated buildings while monitoring for social distancing and sanitizing the surfaces frequently. For those who require computing devices, a loaner program can be implemented where students can borrow laptops for a certain period of time to access the course materials and complete the course requirements. The institution can also provide a virtual desktop environment for students to access all necessary software. Using free scanning applications on smartphones or tablets can address the lack of access to scanners.

Our survey also indicated that about 30% of engineering students had work-life balance issues, while *55%* of students lacked motivation, and 50% did not have access to a private space to attend classes. These results are consistent with those reported in a recent study conducted at Biomedical and Chemical Engineering departments of a Hispanic-serving institution [10]. While the percentage of our students who had issues with lack of motivation or private space seemed to be higher, both studies highlight the necessity of providing more socio-emotional support for students during the difficult times of pandemic.

Students identified various challenges they experienced in online synchronous instruction of courses through Zoom including lack of peer-support/interaction, focus, engagement, and clear guideline from instructors. They also indicated difficulties with time management and Zoom fatigue. Peer-support/interaction has shown to improve the success rate of students especially those from underrepresented groups [24]. Lack of peer-support during the online instruction in the COVID-19 era negatively affects the motivation of the students. However, the remaining raised issues could be addressed in part by employing appropriate teaching techniques by faculty as follows: breaking down a long lecture into shorter segments with more frequent breaks, encouraging group discussion among students, making themselves available during the exams, providing students with a clear roadmap for the online course, making the recordings of the live lectures available after the lecture is over. The latest would help struggling students to learn at their own pace [10]. To assist with the time management issue during the exams, faculty can design practice exams to allow students to familiarize themselves with the questions’ setup and adapt with the exam’s style before the actual exam.

Pandemic has caused educational loss, delayed graduations, cancelled internships and lost job offers. The new generation of students who have been away from face-to-face instructions may lack certain learning experiences. For example, there might be a generation of engineering students who performed the majority of their lab activities virtually and thus, lacks true hands-on skills. While the pandemic educational gap will affect everyone, it is likely to impact under-privileged students (e.g. first generation, low income or care givers) more profoundly [25]. As a result, the socioeconomic factors would constitute key mediators in explaining the potentially large and heterogeneous educational gap. This gap may have long-lasting implications for income inequality and health disparities [26].

To reduce the educational gap, universities could adopt the practice of developing and implementing diagnostic tools to learn where and how large the deficiencies are. Based on the acquired knowledge, they could offer short remediation programs with long-term reorientation of curriculum to align with student’s learning levels [27]. For example, a summer session that deals with hands-on aspects of lab safety or experimentations could be implemented. In some cases, close coordination between the instructors who teach the courses in a sequence may be required, so they can develop extracurricular materials or propose activities that would help students bridge a gap in a specific topic. As the pandemic progresses, the flexibility of university policies could also help with narrowing down the educational gap especially for those students with lower socioeconomical status. Allowing students to adjust their course load, timing of assignments and tuition payment schedule would enable them to make reactive decisions to mitigate the educational loss [25]. A need for further research on this top is undeniable.

### 4.2. Faculty challenges

Establishment of institutional quality standards related to online education is of paramount importance in online education. Effective communication is the key factor in bridging the divide and reconciling administrator and faculty for an enhanced online education [28]. A considerable number of our faculty reported lack of access to hardware, software and necessary tools for online instruction. Especially, in the absence of traditional in-class whiteboard, many faculty members indicated lacking an online writing tool. This issue can be addressed by institution’s budget allocation to acquire necessary hardware and tools (e.g. personal computer/tablet with web camera, digital pen for touch screen devices, digital clipboard, document camera).

Development of online learning assessment methods as rigorous as in conventional face-to-face setting to prevent cheating/plagiarism is not straightforward [16, 29]. While one cannot propose a single assessment method that would work ideally for all engineering courses and classroom sizes, it would still be interesting to study how various online exams and assessment methods (e.g. online quiz tools within the LMS, open-book or take-home examinations, student presentations, peer-reviewed activities, cooperative quizzes [30], oral assessments [31], course summary papers or online portfolios) stack up against each other. Since the onset of pandemic, a limited number of studies (mainly within the fields outside the engineering) have been conducted to evaluate the successes and challenges of the online assessments. The study in [32] revealed that although the majority of undergraduate Management students required more time and effort to prepare for the online exams (compared to the traditional exams), they regarded the clarity and prompt grading and feedback features of the online exams substantially advantageous. Another recent study revealed that cheating remains one of the major concerns for the online examinations and needs to be addressed using available techniques including online proctoring and randomizations of the exam questions [33]. Few other studies showed that the online examinations increased the level of stress and anxiety among medical students [34, 35]. The added stress was in part caused by the lack of a robust examination platform (i.e., reliable LMS) as well as not providing students with sample online practice exams. Finally, a survey conducted among civil engineering students showed that high-achieving students performed significantly better than low-achieving students in online examinations and there was a significant increase in the students’ dropout rate in the 2020–2021 academic year relative to the previous ones [36].

Our student survey results indicated that the use of camera/microphone to proctor the online exams can raise equity concerns (for those who do not have access to camera and cannot afford it) and privacy concern (for monitoring students’ private space). To address these valid concerns, faculty are advised to choose alternative methods for reducing cheating during online exams. Randomizing the exam questions by shuffling both the problem statements and the multiple choices, and randomly selecting a subset of questions from a question library with individualized/randomized input variables are viable practical solutions. Fortunately, most LMS provide these options. However, although 99% of postsecondary US institutions have an LMS in use, only approximately half of faculty at those institutions have been using it on a regular basis [37]. As a result, many faculty members were not familiar with the basic or advances features of the LMS or other tools for effective online instruction. Our survey result confirmed this observation. In fact, our faculty identified a broad range of topics related to BB or other online teaching tools that they felt the need to enhance their skills in. Institutions could address this issue by organizing training workshops, webinars, short-courses, and discussion panels for the faculty to enhance their online teaching skills. At CSULB, stipends were offered in summer 2020 to further incentivize faculty participation in these professional development programs.

Hands-on training is an integral component of engineering education. Following the abrupt conversion of classes to the online format in Spring 2020, many instructors adopted simulations or processing of already acquired data for engineering students to complete their course projects. Our survey indicated the faculty’s need to learn about additional effective ways for providing hands-on training/experience. Depending on the content of the course, employment of “home lab kits” and recording of the lab experiments could partially help. However, design, preparation, distribution/collection of the lab kits or recording of the experiments can be extremely time consuming for faculty especially given all the access restrictions to on-campus labs and additional safety precautions imposed by COVID-19 pandemic. Virtual labs might be a more effective solution. Additionally, remotely accessible labs where the experiment setup is on campus and students use tools for remote control and managing of the setup can be employed, whenever possible [10].

### 4.3. Summary of proposed interventions

From the analysis of the survey results we propose several intervention strategies that can be employed by stakeholders at different levels to improve the online instruction of engineering courses. The proposed strategies (the targeted issues and the survey questions that identified them) are summarized as follows:

➢ Strategies for institution/engineering administration
  - Budget allocation to provide basic equipment for the online instruction to both faculty and students in need. Examples of such equipment include personal computer/tablet preferably with webcam/camera, online writing tool, reliable internet connection (to address the logistical challenges indicated by students and faculty in response to question # 3 of both surveys)
  - Creating a virtual desktop environment and allowing faculty and students to access necessary software (addressing technical access challenges of online instruction indicated in response to questions # 3, #7 and # 11 from the faculty survey, and question #5 from the student survey)
  - Organizing training workshops for faculty/students to further familiarize with online teaching/learning technology and tools (addressing technical skills that were indicated in response to question #10 of the faculty survey and question #5 of the student survey)
  - Providing a syllabus template for online courses including all the important information needed for ABET accreditation (addressing lack of clear communication or instruction indicated in response to question #10 of the faculty survey and question #5 of the student survey)
  - Development and organization of systematic repository of resources pertinent to engineering online instruction (to enhance the faculty’s online teaching skills as the need was indicated in response to questions #10–12 of the faculty survey)
➢ Strategies for engineering faculty
  - Leveraging on the institution’s LMS to manage the course, grades, forum discussions and exams (to enhance the faculty’s online teaching skills as the need was indicated in response to questions #10–12 of the faculty survey)
  - Breaking down a long lecture into shorter segments with more frequent breaks (addressing Zoom fatigue indicated in response to question #4 of the student survey)
  - Encouraging group discussion or problem-solving activities among students such as Zoom breakout rooms (addressing the lack of social interactions with peers as indicated in response to question # 4 of the student survey).
  - Being available during the exams (e.g. on Zoom) to answer students’ questions (addressing the lack of access to the instructors during exams as indicated in response to question # 4 of the student survey).
  - Providing students with a clear roadmap and instruction for the online course (addressing lack of clear communication or instruction indicated in response to question #5 of the student survey)
  - Making the recordings of the live lectures available after the lecture (addressing online instruction challenges and lack of access to reliable internet indicated in response to question #4 and question #3 of the student surveys, respectively)
  - Administering practice exams for students (addressing issues with the online exams indicated in response to question #6 of the student survey)
  - Using open-book/open-note and synchronous assessment methods that support academic integrity. Examples include randomized questions/ restricted time/ question pools on LMS. (addressing the challenges with online assessment methods indicated in response to questions # 4, #7–9 of the faculty survey)
  - Avoiding using camera/microphone to proctor exams (addressing privacy issues with the indicated in response to question #7 of the student survey)
  - Employment of “home lab kits”, recording of the hands-on experiments and virtual labs to partially address the hands-on training aspect of the course (enhancing online instruction as indicated in response to questions # 11–12 of the faculty survey)
➢ Strategies for engineering students
  - Using free scanning applications on their smartphones (addressing lack of access to scanner as indicated in response to questions # 6 of the student survey).

Most of the proposed solutions were implemented at the CSULB college of Engineering in preparation for Fall 2020 semester. Our future work will include evaluation of the efficacy of the implemented interventions by conducting a post-intervention survey at the end of Spring 2021 semester.

This work contributes to the developing body of knowledge about the effect of pandemic on engineering education by investigating the challenges and obstacles faced by a large group of engineering students and faculty at CSULB which exemplifies an institution that previously taught face-to-face engineering classes (predominantly), with a large minority population and socio-economic gap. The recommended strategies for various educational stakeholders (including students, faculty and administration) aims to fill the tools and technology gap, enhance faculty skills in teaching online courses by taking full advantage of online learning management tools, and finally, propose effective assessment methods for online courses while considering the potential equity and privacy issues. These recommendations are practical approaches for many similar institutions around the world and would help improve the learning outcomes of online educations in all fields of engineering.

### 4.4. Potential limitations of the study

Some limitations should be addressed in this study. We investigated the challenges of engineering online education during Spring 2020 – when the pandemic started, and a global emergency occurred. Thus, the reported experiences and perceptions might have been affected by confounding factors related to the onset of pandemic. As the pandemic continues and various academic stakeholders explore and learn about new strategies to better adjust to the new *normal*, subsequent studies conducted in the near future might provide a more accurate picture of the online engineering education.

We advertised the surveys to all faculty and students of the CSULB college of Engineering by sending announcement emails to their university email accounts in summer 2020. While the faculty survey’s response rate was 44%, the student survey’s response rate was 12%. The low response rate of the students might have introduced some participation bias to the results.

Our main goal of conducting the surveys was to identify the urgent needs and challenges of the general body of our students and faculty without focusing on any specific underrepresented groups. Our assumption was that the demographics of survey participants are likely proportional to those of the college of Engineering. Further studies with inclusion of race, gender and socioeconomics demographics are needed to investigate the magnitude of educational challenges that underrepresented groups experienced during the pandemic in comparison with other groups. Consideration of some institutional data (e.g. grades, faculty/ student perception of learning, financial aid requests) from both pre- and during pandemic would enhance the study, as well.

The current work did not evaluate the degree of effectiveness and sustainability of each conducted intervention. It also did not compare the efficacy of various alternative assessment methods for engineering online education. A follow-up study is needed to address these limitations.

## 5. Conclusion

We conducted an observational study to identify challenges encountered due to abrupt transition to online instruction of engineering courses during COVID-19 pandemic by surveying (quantitively and qualitatively) students and faculty at our minority-serving institution. Various logistical, technical and learning/teaching issues were identified, and several interventions were proposed to address them. The results of this study add to the developing body of knowledge about the effect of pandemic on engineering education. This study also provides empirical evidence for the proposed strategies to enhance (and consequently further promote) the online engineering education during and post-pandemic. Our future work will include a thorough study on evaluating the efficacy and sustainability of each proposed intervention.

## Supporting information

S1 Appendix Questionnaire for both student and faculty surveys. (DOCX)

S1 Data Students survey data in response to multiple choice questions. (XLSX)

S2 Data Faculty survey data in response to multiple choice questions. (XLSX)
