# Supplementary material for: An observational study of engineering online education during the COVID-19 pandemic
Source: PLoS One. 2021 Apr 15;16(4):e0250041. doi: 10.1371/journal.pone.0250041 (PMC8049279; doi:10.1371/journal.pone.0250041)
Supplement: S1 Appendix — (DOCX) [file pone.0250041.s001.docx]

8. APPENDIX

Appendix includes copies of both faculty and student surveys.

*8.1.* *FACULTY SURVEY*

1. What is your home department? Select all that apply

a. BME

b. CHE

c. CECEM

d. CECS

e. EE

f. MAE

2. Please select the type of your appointment:

a. Fulltime faculty

b. Part time faculty

3. Check all that you had challenges with (e.g. lack of access or difficulty in operations) in transitioning to online instruction in Spring 2020?

a. Computer and tablet

b. Mic or headset

c. Webcam/camera

d. Scanner

e. Document camera

f. Online writing tools (e.g., digital pen)

g. Printer or cartridge

h. Access to reliable internet (at least 3 Mbps down, 1- 3 Mbps up)

i. Software license

j. VPN and remote access

k. ATS helpdesk and online support

l. Others: Please fill in

4. Which of the following did you primarily use to assess your students’ learning in Spring 2020? Check all that apply.

a. Completely online exams (e.g., BeachBoard Quiz)

b. Asynchronous semi-online exam (download, pen and paper, scan, upload)

c. Synchronous (live) semi-online exam (download, pen and paper, scan, upload)

d. Project/term paper

e. Oral presentation or demonstration

f. Others: Please fill in

5. Which of the following will you primarily use to assess your students’ learning in Summer/Fall 2020? Check all that apply.

a. Completely online exams (e.g., BeachBoard Quiz)

b. Asynchronous semi-online exam (download, pen and paper, scan, upload)

c. Synchronous (live) semi-online exam (download, pen and paper, scan, upload)

d. Project/term paper

e. Oral presentation or demonstration

f. Others: Please fill in

6. What kind of classes will you be teaching in Summer/Fall 2020? Select all that apply.

a. Lecture only.

b. Lecture with non-hands-on lab or activity, e.g., simulation and problem solving, etc.

c. Lecture with hands-on lab or activity

d. Others (a type of class not listed above, or being the course coordinator): Please fill in

7. If you had an online exam (semi- or completely), which type was it? Check all that apply.

a. Open book/notes

b. Closed book/notes

c. Exam that requires the use of specific software

d. Other (fill in)

8. If you had an online exam, how did you proctor it? Check all that apply.

a. Using Zoom camera and mic on

b. Using Lockdown browser and Respondus monitor

c. Used online exams, but did not proctor it

d. Other (fill in)

9. What is your perception of the extent of cheating/plagiarism in Spring 2020 relative to prior semesters?

a. Way less

b. Less

c. About the same

d. More

e. Way more

f. I do not know/ Did not use online exam

g. Other (fill in)

10. Indicate all your topics of interest to enhance your skills (by either attending a workshop or watching a webcast).

a. Online course syllabus: What are the requirements of an online course syllabus

b. Basic BeachBoard (BB) features: How to create/modify/improve BB for my course

c. More advanced BB features: How to create online surveys /discussion groups/quizzes that reduces the potential of cheating, how to automatically export grades to BB Grades, how to use Master Shell in BB, etc.

d. Zoom features (basic and advanced): How to schedule/record a meeting, how to use Zoom’s Whiteboard or OneNote, how to do breakout rooms, etc.

e. Multimedia skills: How to create interactive multimedia files using Kaltura Capture, Camtasia or Snagit, how to use Alt captions in media you generate (Word, PPT, page in BB) to facilitate accessibility

f. Assessment: How to do automatic grading using software that helps grading (e.g. Gradescope)

g. Learning Objective (LO): What are student Los and module LOs? How to align them?

h. Other: to be filled

11. If you were to be provided with a personal trainer, what are your top two online teaching challenges that you would like the trainer to help you with for teaching your class more effectively in Summer/Fall 2020?

12. Please provide any additional comments here on the challenges you have faced regarding teaching and issues that we might be able to help you resolve in Summer/Fall 2020.

*8.2.* *STUDENT SURVEY*

1. What is your major? (select all that apply)

a. Biomedical Engineering

b. Chemical Engineering

c. Civil Engineering

d. Construction Management Engineering

e. Computer Engineering

f. Computer Science

g. Electrical Engineering

h. Mechanical Engineering

i. Aerospace Engineering

j. Engineering Technology

2. What’s your academic level?

a. Freshman (mainly taking 100-level courses within your department)

b. Sophomore (mainly taking 200-level courses within your department)

c. Junior (mainly taking 300-level courses within your department)

d. Senior (mainly taking 400-level courses within your department)

e. Graduate level

f. Other: please fill in

3. Which of the following will be a challenge for you when taking classes in a fully online environment? Check all that apply

a. No access to a computer

b. Sharing computer with others

c. No access to a computer with camera/ webcam

d. No internet access

e. No reliable/high speed internet access

f. No private/quiet space to work in

g. Working to support myself or my family, therefore not enough time to study

h. Time management, Lack of motivation

i. Others: Please fill in

4. Which of the following challenges did you experience with Zoom synchronous (live) classes in Spring or Summer 2020?

a. Did not have reliable internet connection

b. Was not able to focus and follow lectures

c. Felt socially disconnected from peers

d. Felt Zoom fatigues (overwhelmed with multiple online sessions)

e. Did not feel engaged

f. Others: Please fill in

5. Which of the following challenges did you experience with classes taught in online instruction mode in Spring or Summer 2020? Select all that apply

a. No clear instructions

b. Lack of communication

c. Issues with accessing course material

d. Issues with using technology/software

e. Navigating BeachBoard to access assignments/exams

f. Issues with submitting assignments

g. Others: Please fill in

6. Which of the following challenges did you experience with online exams? Select all that apply

a. Time management

b. No access to a quiet space to take the exam

c. No access to a printer/scanner to print out or submit the exam

d. No access to a reliable internet

e. No access to instructors during exams

f. Exams were more difficult than in-class exams

g. Issues with methods of proctoring exams, such as: Respondus Lockdown Browser, Zoom, etc.

h. Noticed more cheating among classmates

i. Others: Please fill in

7. Do you feel comfortable using a cell phone or computer camera and showing your face, only for the purpose of identification during demos, presentations, or exams?

a. Yes

b. No

c. I cannot because I don’t have a camera

d. Other concerns: Please fill in

8. How would you rate your overall experience with taking classes in an online mode of instruction in Spring 2020?

a. Very satisfied

b. Satisfied

c. Neither satisfied nor dissatisfied

d. Dissatisfied

e. Very dissatisfied
